# STATs in NK-Cells: The Good, the Bad, and the Ugly

**DOI:** 10.3389/fimmu.2016.00694

**Published:** 2017-01-18

**Authors:** Dagmar Gotthardt, Veronika Sexl

**Affiliations:** ^1^Department for Biomedical Sciences, Institute of Pharmacology and Toxicology, University of Veterinary Medicine, Vienna, Austria

**Keywords:** JAK–STAT, tumor surveillance, cytotoxicity, immunologic, mouse models, NK cells, VEGF-A, tumor promotion

## Abstract

Natural killer (NK)-cells are major players in the fight against viral infections and transformed cells, but there is increasing evidence attributing a disease-promoting role to NK-cells. Cytokines present in the tumor microenvironment shape NK-cell maturation, function, and effector responses. Many cytokines signal *via* the Janus kinase (JAK)–signal transducer and activator of transcription (STAT) pathway that is also frequently altered and constitutively active in a broad range of tumor cells. As a consequence, there are currently major efforts to develop therapeutic strategies to target this pathway. Therefore, it is of utmost importance to understand the role and contributions of JAK–STAT molecules in NK-cell biology—only this knowledge will allow us to predict effects of JAK–STAT inhibition for NK-cell functions and to successfully apply precision medicine. We will review the current knowledge on the role of JAK–STAT signaling for NK-cell functions and discuss conditions involved in the switch from NK-cell tumor surveillance to disease promotion.

## Introduction

Natural killer (NK) cells are major players of the innate immune system and immediate effector cells against viral infections, pathogens, and malignant cells. In humans, NK-cells compromise 5–15% of circulating blood lymphocytes and are further sub-divided based on the expression of the cell adhesion molecule CD56 and the low affinity Fc-receptor CD16 into CD3^−^CD56^bright^CD16^−^ and CD3^−^CD56^dim^CD16^+^ NK-cells. CD56^bright^ NK-cells are mainly found in lymph nodes, produce cytokines upon activation, and possess only minor cytotoxic potential. Upon maturation to CD56^dim^ cells—the majority of circulating NK-cells in healthy humans representing approximately 90% of NK-cells—gain significant cytotoxic potential (1, 2). There is ample evidence for the ability of NK-cells to recognize and lyse a broad variety of tumor cells (3–5). The ability of NK-cell-mediated immune surveillance extends to the prevention of metastatic spread (6–8), which is currently one of the dominating clinical problems in cancer therapy.

Initially described to function without prior sensitization, accumulating evidence demonstrates that NK-cell effector function is a complex and tightly regulated process (9–12). This ensures rapid effector reactions while preventing autoimmunity (13). During development, NK-cells undergo a licensing process that shapes their responsive steady state. NK-cells lacking inhibitory receptors or unable to recognize cognate MHC molecules maintain a hyporesponsive state (13, 14). Even if fully developed and equipped to act against target cells, NK-cells require a costimulatory signal to pull the final trigger. Cytokines provided by the microenvironment or the ligation of activating receptors serve as promoting signals (15, 16). NK-cell activation is thus also controlled by the availability of cytokines including type I interferons (IFNs) from TLR^+^ cells. IFNAR signaling in dendritic cells leads to the subsequent production of IL-15 that is trans-presented to activate NK-cells (17). Besides IL-15, also IL-2 produced by CD4^+^ T cells stimulates NK-cell activation while regulatory T cells (T_regs_) inhibit NK-cell responses in a TGF-β-dependent manner. Moreover, T_regs_ expressing the high affinity IL-2 receptor alpha chain CD25 limit the availability of IL-2 for NK-cells (18, 19).

Another layer of complexity is added by the escape mechanisms of tumor cells. Tumor cells evade NK-cell recognition by several mechanisms including changes in expression of MHC class I or secretion of cytokines and mediators impeding NK-cell responses (20). Immunosuppressive cytokines such as TGF-β or adenosine in the tumor microenvironment block NK-cell maturation and their cytotoxic potential or act indirectly by recruiting suppressor cells (21, 22). Cytokines have thus both abilities; they may activate or block NK-cells.

Most cytokines influencing NK-cell functions signal *via* Janus kinase (JAK)–signal transducer and activator of transcription (STAT) pathway, a conserved pathway transmitting extracellular signals from the cell surface to the nucleus (23). The JAK–STAT pathway is frequently altered and constitutively active in a broad range of tumors. There are major efforts to develop therapeutic strategies to target components of this pathway (24–26). It is thus critical to comprehend the role of JAK–STAT molecules in NK-cell biology. This knowledge will enable to predict effects of JAK–STAT inhibition for NK-cells, a prerequisite for precision medicine.

## JAK–STAT

Cytokine binding to a respective receptor on the cell surface leads to the activation of receptor-associated tyrosine kinases, the JAKs. Once activated, JAKs trans-phosphorylate each other, thereby creating docking sites for signal transducer and activator of transcription (STAT) molecules. Subsequent to binding, STATs become activated by JAK-mediated tyrosine phosphorylation and form homo- or heterodimers, translocate to the nucleus where they regulate transcription (27, 28). Four distinct JAK kinases (JAK1, 2, 3, and TYK2) as well as seven different STAT proteins exist (STAT1, 2, 3, 4, 5A, 5B, and 6). One cytokine may activate more than one member of the JAK and/or STAT family (29). Table 1 summarizes our current knowledge on JAK–STAT signaling in NK-cells.

**Table 1:** **Janus kinase (JAK)/signal transducer and activator of transcription (STAT) signaling in natural killer (NK)-cells (27, 30–45)**.

Cytokine	Receptor-associated JAKs	Activated STATs	Function	Effect induced by
IL-2	JAK1, JAK3	STAT1, STAT3, STAT5	Proliferation	STAT5
	JAK2	STAT4	Activation	STAT1/4/5; STAT3?
IL-7	JAK1, JAK3	STAT5	Survival of CD56^bright^ NK-cells, upregulation of FasL	STAT5
			Development of distinct NK-cell subsets	
IL-12	JAK2, TYK2	STAT1, STAT3, STAT4	Activation	STAT1/4
			Induction of *Vegf-A* expression	STAT3?
IL-15	JAK1, JAK3	STAT5	Survival, maturation, proliferation	STAT5
		STAT3	Activation	STAT5, STAT3?
IL-10	JAK1	STAT3	Activation	STAT3
			Induction of *Vegf-A* expression	STAT3?
IL-21	JAK1, JAK3	STAT1, STAT3	Antiproliferative (mouse NK-cells), proliferation (human NK-cells)	STAT3?
			Maturation, activation	STAT1?
			Induction of *Vegf-A* expression	STAT3?
IL-27	JAK1	STAT1, STAT3, STAT5	Activation	Unknown
			Increased ADCC	STAT5?
			Increased IL-10 production	STAT3?
			Increased viability	STAT5?
			Decreased proliferation	STAT3?
Interferon-α/β	JAK1, TYK2	STAT1, STAT3	Maturation	STAT1; STAT4?
			Activation	STAT1/3/4
			Induction of *Vegf-A* expression	STAT3?

## JAKs: The Driver of the STATs

One cytokine may activate more than one JAK and each JAK targets more than one STAT protein. This multilayered and complex activation pattern creates sometimes elaborate phenotypes upon deletion or inhibition of single components (46). The distinct roles of JAK kinases for NK-cell biology are on the edge of being unraveled, currently only limited information is available.

Treatment with the JAK1/JAK2 inhibitor ruxolitinib reduces NK-cell numbers, impairs their proliferation, maturation, and cytolytic capacity. Application of ruxolitinib in a murine breast cancer model enhanced metastatic spread by interfering with NK-cell functions (7, 47). The fact that ruxolitinib efficiently inhibits JAK1 and JAK2 but also with low affinity JAK3, makes it difficult to assign specific roles to distinct members of the JAK family. NK-cells fail to develop in *Jak3*^−/−^ mice—a phenotype that is mirrored in patients harboring *Jak3* mutations. These patients suffer from a SCID phenotype lacking T and NK-cells (48–50). The contribution of JAK1 and JAK2 on NK-cell development and function needs to be further explored. While JAK3 is predominantly expressed in the hematopoietic compartment, JAK1 and JAK2 are ubiquitously expressed and *Jak1* and *Jak2* knockouts are perinatal/embryonic lethal (51, 52). JAK1 has been reported to be crucial for lymphopoiesis, and both JAK1 and JAK3 are important upstream kinases mediating IL-15-dependent signaling and subsequent STAT5 activation (52–54). It is attractive to speculate that loss of JAK1 would as well induce the loss of peripheral NK-cells.

Experiments using *Jak2*^−^ conditional knockout mice uncovered a critical role for JAK2 in NK-cell maturation (7). Breast cancer metastasis related to impaired NK-cell function was enhanced in mice treated with the JAK2-specific inhibitor BSK805. Simultaneous treatment with IL-15 prevented the enhanced metastasis provoked by JAK2 inhibition. This indicates that BSK805-mediated JAK2 inhibition does not affect IL-15-mediated responses in NK-cells presumably acting *via* JAK1 and JAK3 (7). Only the generation and analysis of NK cell-specific conditional knockout mice will allow us to characterize the individual effects of JAKs on NK-cell development and effector function.

In contrast to other JAKs, *Tyk2*^−/−^ NK-cells are present at normal numbers but show impaired IL-12/IL-18-mediated signaling with reduced STAT4 activation. Consequently, *Tyk2*^−/−^ NK-cells possess a severely impaired cytolytic activity, do not efficiently clear certain infections, and display an impaired tumor immune surveillance (55–58). In line, patients with autosomal recessive *Tyk2* mutations suffer from recurrent bacterial and viral infections and display impaired NK-cell responses (59).

## The Good: STAT1: It Turns the Killing on

STAT1 and STAT2 are well studied transcription factors and important for signals in response to IFNs (60). Our knowledge on STAT2-regulated NK-cell functions is limited; it is known that STAT2 controls viral load during LCMV infections (61). In contrast, STAT1 effects have been characterized in more detail. STAT1 is a crucial regulator of IFN-γ production and NK-cell cytotoxicity (60–62). *Stat1-*deficient mice are highly susceptible to bacterial and viral infections. *Stat1*^−/−^ mice show reduced expression of MHC class I molecules, which is thought to lead to hyporesponsive, unlicensed NK-cells (63, 64). It is currently unclear whether the impaired cytotoxicity is solely the consequence of the impaired licensing or whether STAT1 fulfills other major functions. The complexity of STAT1 signaling in innate immunity is further highlighted by the existence of a non-canonical STAT1 pathway. STAT1^Y701F^ mutant proteins that cannot be activated by JAKs in the canonical manner partially rescue impaired cytolytic responses of *Stat1*^−/−^ NK-cells. One potential explanation for this unexpected phenomenon is the finding that STAT1 locates to the immunological synapse when NK-cells conjugate target cells. In line, STAT1 has been shown to bind proteins involved in cell junction formation at the immunological synapse during tumor cell recognition (65). Moreover, *ex vivo* derived NK-cells show a constitutive phosphorylation of the STAT1-S727 residue restraining NK-cell cytotoxicity. This phosphorylation is present without any stimulus and prior to tyrosine phosphorylation, thus deviating from the canonical STAT activation (6, 28). These observations point at a complex and multilayered function of STAT1 in NK-cells and suggest STAT1 as a central node integrating several processes.

Many effects described in *Stat1*-deficient mice are mirrored in patients. STAT1 deficiency in humans is an autosomal recessive immune disorder; null mutations are associated with recurrent bacterial and viral infections indicating impaired NK-cell activities although no detailed information is available so far (66–70).

## The Ugly: STAT3: Avoiding Autoimmunity or the Target for NK-Cell Therapy?

While cytokines such as IL-12, IL-15, IL-21, and type I IFNs induce STAT3 tyrosine phosphorylation in NK-cells, the most potent activation is achieved by treatment with the immunosuppressive and anti-inflammatory cytokine IL-10 (71). Many tumors harbor constitutively active STAT3 that triggers the release of immunosuppressive cytokines such as IL-10 or TGF-β. These tumor-derived cytokines further induce a pronounced STAT3 phosphorylation in infiltrating immune cells. There, induced STAT3 activation is considered to impair tumor immune surveillance and allows the tumor to escape immune control (72, 73). High levels of STAT3 phosphorylation in the tumor stroma often correlate with loss of intact tumor immune surveillance (74). This effect is of therapeutic interest as STAT3 inhibitors are currently developed to treat patients suffering from cancer of various origin (75, 76). There is dual hope in these STAT3-directed therapies; on the one side, they are expected to block STAT3-mediated growth promoting and pro-survival signals in the tumor cells themselves. On the other hand, STAT3 inhibitors directly act on the infiltrating immune cells and might boost their cytotoxic behavior.

There is first evidence that this concept holds true for NK-cells. Studies in mouse models uncovered that STAT3 activation in NK-cells indeed suppresses cytotoxicity. The deletion of STAT3 in NK-cells enhanced cytotoxicity in melanoma and leukemia models (71, 77) and resulted in a prolonged survival (71). The absence of STAT3 was paralleled by an increased expression of perforin and granzyme B and the activating receptor DNAM-1. There is conflicting evidence if and how STAT3 also regulates the expression of the activating NKG2D receptor in NK-cells. In human, NK-cells stimulation with IL-10 and IL-21 induces NKG2D expression in a STAT3-dependent manner. Similar results were obtained in a mouse study showing enhanced NKG2D-mediated antitumor responses upon IL-21 treatment (78, 79). Against the expectations, *Stat3*^−/−^ NK-cells isolated from *Stat3^fl/fl^Ncr1*-Cre^Tg^ mice, where deletion of STAT3 is restricted to NKp46^+^ cells, show no changes in NKG2D expression (71). In contrast, NK-cells analyzed from *Stat3^fl/fl^VavCre* mice showed reduced NKG2D expression (79). The controversy is further heated by a study showing that IL-21 stimulation inhibits NKG2D expression of IL-2-cultured primary human NK-cells (80). Several scenarios may explain these conflicting results; one may envision that STAT3 is involved in epigenetic processes that control NKG2D expression and that occur prior to NKp46 expression. In such a scenario, the deletion of STAT3 in a NKp46^+^ population would be too late in NK-cell development to interfere with NKG2D expression. Alternatively, the regulation of NKG2D expression in NK-cells might require cell extrinsic-cues that depend on STAT3 and are lost in *Stat3^fl/fl^VavCre* mice upon deletion in the entire hematopoietic system (79).

Of note, STAT3 inhibition in tumors has been shown to enhance immunogenicity even in tumors that do not depend on STAT3 for survival and growth. One of the mechanisms how immunogenicity is increased is the enhanced expression of NKG2D ligands on tumor cells (81, 82).

Another consequence of STAT3 deletion in NK-cells is an increased expression level of STAT5 (71). As described below, STAT5 is a potent stimulator of NK-cell survival and cytotoxicity. It remains to be determined how any STAT3-directed therapy will interfere with the delicate balance of STAT3-mediated suppression and STAT5-mediated activation of NK-cell cytotoxicity. This is of particular relevance when employing cytokines that act *via* both STAT proteins, e.g., IL-15. It is attractive to speculate that IL-15-induced STAT3 activation may serve to counteract the IL-15-STAT5-mediated NK-cell cytotoxicity to prevent autoimmunity. A detailed understanding of the mechanisms governing the repression of NK-cell overshoots is of utmost therapeutic importance. Cancer therapies aim at increasing the potential of killers while avoiding self-destruction.

## The Good: STAT4: You Better have More

STAT4 is a prerequisite for IL-12-mediated cytotoxicity and IFN-γ production in murine and human NK-cells (83, 84). Additionally, STAT4 has been described to induce T-bet and IL-10 in NK-cells and to be involved in the generation of memory NK-cells after MCMV infection (83, 85, 86). Direct binding of STAT4 to the perforin promoter has been reported in human NK-cells (87). Besides its potent activation by IL-12 stimulation, high basal levels of STAT4 protein expression have been detected in murine and human NK-cells (61). In contrast to other immune cells, IL-2 treatment activates STAT4 in NK-cells and enhances responses to IL-12 by upregulating of the IL12R (38, 84). It is attractive to speculate that the constitutively high expression levels of STAT4 represent a “ready-to-go” repertoire that enables NK-cells to immediately react on cytokine exposure. This hypothesis is supported by the fact that NK-cells represent the first line of defense against pathogens—their rapid and efficient activation being a prerequisite. In line, tolerogenic NK-cells have been reported in the context of liver transplantation, where immunosuppression subsequently decreased STAT4 levels and resulting in hyporesponsive NK-cells (88).

Although IL-12 possesses the potential to also activate STAT1 and STAT3, STAT4 appears to be crucial in mediating IL-12-induced signaling and IFN-γ production. The role of IL-12-induced STAT1 and STAT3 activation for IFN-γ production is currently unclear. It may represent an evolutionary backup to induce a second wave of IFN-γ response. On the other hand, STAT1 and STAT3 may act as feedback loop and prevent successive production. In fact, binding of several STAT molecules to the IFN-γ promoter has been reported (71, 89).

## The Good: STAT5: Teaches NK-Cells How to Drive

STAT5 transmits signals downstream of IL-2 and IL-15, and its expression is indispensable for the survival of peripheral NK-cells (90). STAT5 exists of two homologs, STAT5A and STAT5B, that share more than 90% sequence identity and arose by gene duplication (91). There is evidence that the loss of STAT5B, but not STAT5A reduces NK-cell numbers and impairs cytolytic responses (92). This is mirrored in patients harboring *Stat5b* deficiencies and suffering from NK-cell lymphopenia, recurrent bacterial and viral infections, several clinical pathologies, and high morbidity (67, 93). While the deletion of STAT5B only reduces NK-cell numbers to 50%, the targeted deletion of STAT5A and STAT5B in NK-cells induces apoptosis and leads to a complete loss of peripheral NK-cells (90). These data indicate that both STAT5 isoforms are involved in NK-cell maturation and survival (90). Survival of STAT5-deficient NK-cells can be rescued by the enhanced expression of the anti-apoptotic gene *Bcl-2* and allows studying the role of STAT5 for other NK-cell functions. STAT5 is not only regulating NK-cell survival, proliferation, and cytotoxicity but also drives cell maturation (94) by driving the expression of transcription factors involved in NK-cell maturation and survival (94). Besides allowing NK-cell maturation and cytotoxicity, STAT5 suppresses the tumor-promoting potential of NK-cells (94). Similar to myeloid cells, NK-cells have the potential to support tumor growth by secreting VEGF-A (94, 95). VEGF-A expression and thus tumor promotion is suppressed by STAT5 with STAT5B being the relevant isoform (94). There is accumulating evidence for the existence of VEGF-A secreting tumor infiltrating NK-cells in patients suffering from small lung cell cancer, breast, and colon tumors (96, 97). These tumor-promoting NK-cells are immature (CD56^bright^), and their presence has been correlated to poor disease prognosis in several studies (98–100). Therefore, it is attractive to speculate that IL-2- and IL-15-mediated STAT5 activation in cancer patients does not only activate NK-cell cytotoxicity but also reverts pro-angiogenic effects. Decidual NK-cells have been the first NK-cells reported to produce VEGF to promote trophoblast invasion and remodeling of spiral arteries (101–103). Uterine NK-cells are poorly cytotoxic with a particular cytokine profile (101). It remains to be elucidated whether STAT5 is also involved in VEGF-A production in the decidua. A suppressive cytokine milieu such as TGF-β in the uterus or hypoxic conditions might dampen STAT5 signaling and represent a prerequisite for VEGF-A transcription. Evolution brought two types of NK-cells into light: besides being effective killers NK-cells have acquired to adapt to immunosuppressive cytokines and to switch to a tolerogenic but pro-angiogenic behavior.

## The Bad: STAT6: Still Some Missing Bricks

Activation of STAT6 has been reported to drive IL-5 and IL-13 production in cultured NK-cells and to limit cytotoxic responses (104). In line, studies with *Stat6*^−/−^ mice showed increased viral resistance and higher cytolytic activity of NK-cells in the absence of STAT6 (105). However, a positive correlation of STAT6 expression and IFN-γ production was reported after costimulating murine NK-cells with IL-4 and IL-2 (106). Further studies need to explore whether a STAT6 blockade would be a potential therapeutic option to enhance responses in human NK-cells.

## Conclusion

**Figure 1:**
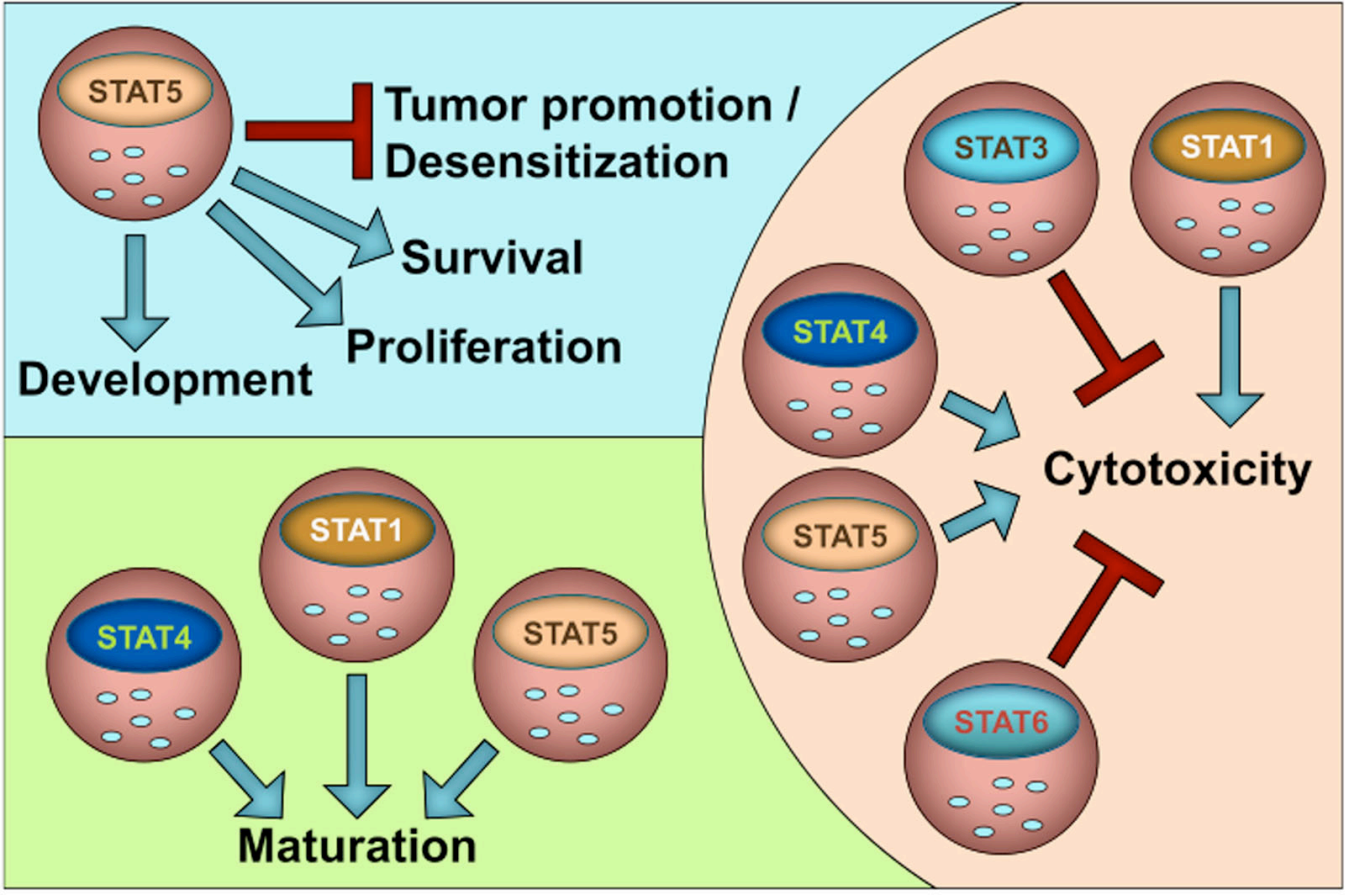
**The role of STATs for NK cell homeostasis and function**.

The JAK–STAT pathway is evolutionary highly conserved; thus, the human situation nicely matches the findings in experimental animal models. In that line, many insights that we gained from murine NK-cells can be translated to human NK-cells. Figure 1 summarizes our current knowledge on the role of STATs in NK-cell functions. In general, STAT1, STAT4, and STAT5 stimulate NK-cell maturation and cytotoxicity, whereas STAT3 and STAT6 negatively impact on NK-cell activity. It is attractive to speculate that the suppressive role of STAT3 and STAT6 is important to prevent NK-cell overshoots and autoimmunity. STAT5 is the only STAT family member that is indispensable for NK-cells since it governs survival and growth in addition to cytotoxicity and maturation. It may thus be seen as NK-cell master regulator.

As shown for macrophages NK-cells not only inhibit but also promote tumor formation, e.g., by producing VEGF-A. So far, STAT5 has been shown to prevent NK-cell-mediated tumor promotion by suppressing VEGF-A. However, it is unclear if and how other family members contribute to the switch from tumor suppression to tumor progression. Another layer of complexity is added by the fact that STATs rarely act alone but are embedded in a network of signaling events depending on the microenvironment and stimuli present. Signal integration is required to determine outcomes; at its lowest level integration of activity arising from various STAT family members is needed as even a single cytokine can activate multiple STATs (listed in Table 1). Some cytokines activate STAT family members with opposing functions such as IL-12 or type I IFNs. Further research will have to link NK activity and biological outcomes to cytokine-induced STAT activation and their synergic and/or antagonistic roles. The evolving field of systems biology may be of help to address these issues and/or to even predict the complex biologically and medically relevant questions *in vivo* at high pace to optimize current cancer therapies.

## Author Contributions

All authors listed have made substantial, direct, and intellectual contribution to the work and approved it for publication.

## Conflict of Interest Statement

The authors declare that the research was conducted in the absence of any commercial or financial relationships that could be construed as a potential conflict of interest.
